# CO_2_ Uptake of Carbonation-Cured Cement Blended with Ground Volcanic Ash

**DOI:** 10.3390/ma11112187

**Published:** 2018-11-05

**Authors:** Joon Ho Seo, Issam T. Amr, Sol Moi Park, Rami A. Bamagain, Bandar A. Fadhel, Gwang Mok Kim, Ali S. Hunaidy, Haeng Ki Lee

**Affiliations:** 1 Department of Civil and Environmental Engineering, Korea Advanced Institute of Science and Technology, Daejeon 34141, Korea; junhoo11@kaist.ac.kr (J.H.S.); solmoi.park@kaist.ac.kr (S.M.P.); 2 Carbon Management Division, Research & Development Center, Saudi Aramco, Dhahran 31311, Saudi Arabia; issam.amr@aramco.com (I.T.A.); rami.bamagain@aramco.com (R.A.B.); bandar.fadhel@aramco.com (B.A.F.); ali.hunaidy@aramco.com (A.S.H.); 3 Korea Institute of Geoscience and Mineral Resources, Daejeon 34132, Korea; k.gm@kigam.re.kr

**Keywords:** Saudi volcanic ash, natural pozzolan, carbonation, Portland cement, CO_2_ uptake

## Abstract

Accelerated carbonation curing (ACC) as well as partial replacement of cement with natural minerals are examples of many previous approaches, which aimed to produce cementitious products with better properties and environmental amicabilities. In this regard, the present study investigates CO_2_ uptake of carbonation-cured cement blended with ground Saudi Arabian volcanic ash (VA). Paste samples with cement replacement of 20%, 30%, 40%, and 50% by mass were prepared and carbonation-cured after initial curing of 24 h. A compressive strength test, X-ray diffractometry (XRD), and thermogravimetry were performed. Although pozzolanic reaction of VA hardly occurred, unlike other pozzolana in blended cement, the results revealed that incorporation of VA as a supplementary cementitious material significantly enhanced the compressive strength and diffusion of CO_2_ in the matrix. This increased the CO_2_ uptake capacity of cement, reducing the net CO_2_ emission upon carbonation curing.

## 1. Introduction

Increasing global CO_2_ emission has been posing threats to the Earth’s atmospheric environments. It was reported that the annual CO_2_ concentration at the Earth’s surface in 2017 reached 405 ppm, which is approximately quadruple to that in the early 1960s [1]. The cement industry and the production of Portland cement (PC) embody 5–8% of global CO_2_ emissions, which is one of the major greenhouse gases contributing to global warming [2,3]. Numerous attempts have been identified as potential means to reduce the CO_2_ associated with the production of cement, such as (1) alternative clinkers, including reactive belite-rich PC and belite-ye’elimite-ferrite cement, which have less limestone input and lower sintering temperature [4,5], alkali-activated binders derived from alkaline activation of aluminosilicate precursors sourced from industrial by-products or natural pozzolans [6,7,8,9], and phosphate-derived hydraulic binders, including both calcium and magnesium phosphate cement [10]; (2) PC clinker substitution with supplementary cementitious materials (SCMs), which are industrially sourced or naturally abundant [11,12]; and (3) accelerated carbonation curing (ACC), in which CO_2_ generated from various industries are collected and are utilized [13,14,15]. As the removal of emitted CO_2_ is a serious concern, numerous attempts have been made to stabilize gaseous CO_2_ by carbon sequestration under the theme of reusing CO_2_ with ACC technologies [16,17].

ACC technology for concrete production has received significant attention because it does not only facilitate storing a large amount of CO_2_, but also has a general tendency to improve the mechanical properties [13,14,18]. In particular, the energy embodied for the production of 1 m^3^ of concrete is ~0.6 GJ using steam curing, while a similar strength of concrete can be obtained by 0.013 GJ using carbonation curing utilizing industrial flue gas balanced with 20% CO_2_ [19,20]. The potential CO_2_ reduction materialized by carbonation curing is the storing of CO_2_, as well as lower input energy during the manufacture of precast concrete [21]. The strength development of concrete during carbonation curing is accelerated by carbonation-induced reaction of belite, which has a lower hydraulic activity than alite, and precipitation of calcium carbonate [14]. These two phenomena occur relatively easily when the concrete is in the fresh state, since the diffusion of gaseous CO_2_ is easier than it would be into the concrete in the hardened state [14].

Substitution of cement with SCMs is another effective and straightforward means of reducing the CO_2_ footprint of concrete, and is also preferred in many cases around the world for economic benefits, which stem from the lower cost of SCMs than that of PC [11]. Those that are typically used in practice and intensively investigated among the scientific communities are PC blended with silica fume [22,23], fly ash [24,25,26], and slag [27,28]. The calcium-silicate-hydrate (C–S–H) present in blended PC is generally agreed to resemble a tobermorite-like structure, and typically has a lower Ca/Si ratio, which leads to higher Al uptake [11,29]. The use of SCMs in concrete production is increasing, i.e., the average amount of clinker in cement was 85% in 2003 and 77% in 2010, while the annual worldwide production and consumption of cement is also increasing [12]. Currently, ground-granulated blast furnace slag and fly ash are the two representative SCMs used, however, the supply of these materials will be difficult to meet the demand of cement given that the current usage of SCMs is to be maintained. In some parts of the world, the energy policy is shifting toward developing renewable and sustainable sources, and abandoning traditional coal-fired power generation, meaning that fly ash may not be generated in the near future [30]. Moreover, most oil-producing countries rely on diesel generators, therefore, typical industrially-sourced pozzolans are not available. A way to meet the demand of cement while maintaining the current usage of SCMs is to embrace other pozzolanic materials, such as naturally occurring minerals.

Volcanic ash (VA) is a fragmented rock consisting of glassy minerals, abundantly available in a large quantity in the world, especially in the western region of Saudi Arabia with an approximate area of 90,000 km^2^ [31], and other neighboring countries, and is rich in Si and Al. Numerous studies were conducted to assess the effect of VA incorporation on the performance of concrete, along the line of studies of high volume fly ash concrete [32]. These studies investigated VA for use as a mineral admixture in concrete in a similar manner to Class F fly ash [33]; blended cement incorporating VA was found to have better durability performance, such as chloride penetration and fire resistance, while replacement of above 20% may lead to a decline in the mechanical strength [34,35].

Numerous studies were conducted to investigate the carbonation behavior of PC blended with SCMs [36,37,38,39]. On the one hand, SCMs incorporated in PC effectively consumes Ca(OH)_2_ and forms C–S–H, which is not fully carbonated, leading to a lessert amount of CaCO_3_ being formed; however, it should be noted that the depth of carbonation generally increases as a higher amount of SCM is used to replace PC [39]. In this context, incorporation of SCM in PC may lead to a reduction of the footprint of CO_2_ in concrete production by (1) reducing the use of clinker; and (2) allowing carbonation to occur at a faster rate. The present study investigates the CO_2_ uptake capacity of carbonation-cured PC blended with VA. PC paste samples were synthesized by substituting PC with VA by 0%, 20%, 30%, 40%, and 50% by mass of cement. The samples were carbonation-cured by exposing them for one day in an accelerated carbonation chamber at a 5% atmospheric CO_2_ concentration. The effects of carbonation-curing and VA incorporation are explored using multiple analytical techniques, and are discussed in the context of developing green concrete.

## 2. Experimental Program

### 2.1. Materials and Sample Preparation

**Figure 1:**
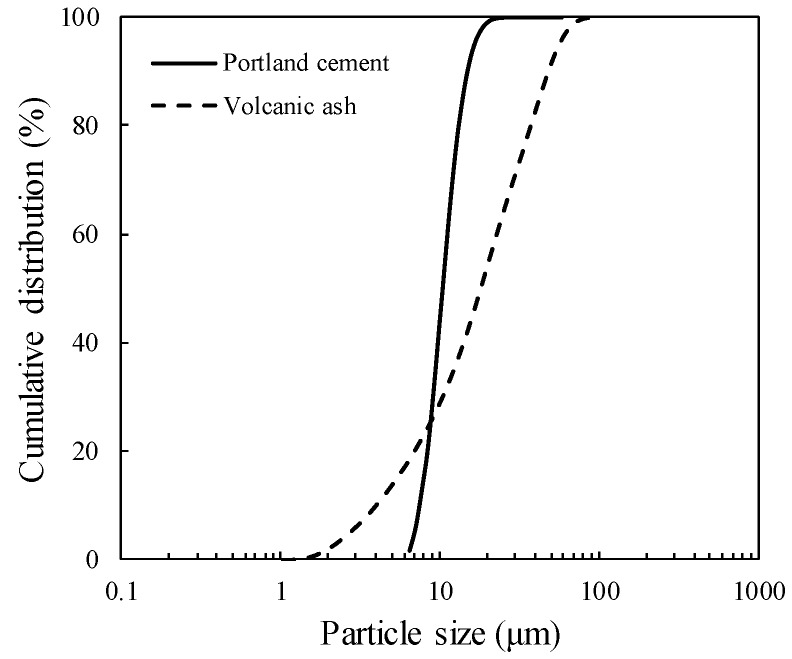
Particle size distribution of Portland cement and volcanic ash.

**Figure 2:**
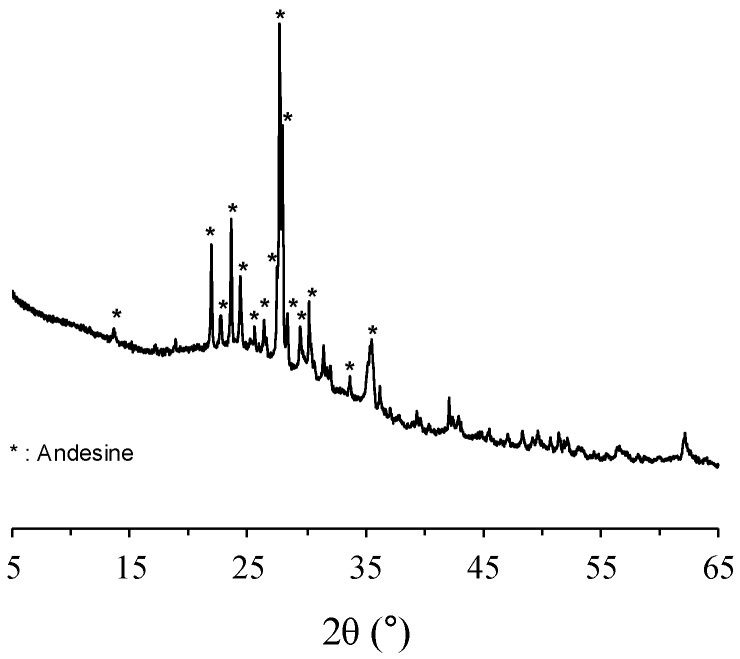
X-ray diffractometry (XRD) pattern of the volcanic ash distribution of volcanic ash.

PC conforming to Type I of ASTM C150 was used in all mixtures. The PC was produced from Ssangyoung Cement Industrial Co., Ltd. (Seoul, Korea) and its chemical composition is provided in Table 1. The VA obtained from Harrat Rahat, western region of Saudi Arabia, southern part of Al-Madina city, was used to partially substitute PC to achieve binary binding materials. The VA was ball-milled for an hour to achieve the particle size distribution, as shown in Figure 1. Note that the median powder value of ball-milled VA was 19.84 μm. The chemical compositions of the VA used in this study can be found in Table 2. The mineralogical constituent of the VA mostly consisted of plagioclase feldspar (andesine (Al_1.488_Ca_0.491_Na_0.499_O_8_Si_2.506_, #PDF 01-079-1148)) with an amorphous hump in the region of 20–30° 2*θ* (Figure 2). It was calculated from the quantitative analysis of X-ray diffractometry (XRD, Malvern Panalytical, Malvern, UK) pattern that 65.1% of VA is composed of the amorphous glassy phase.

The samples were made with a constant water-to-powder ratio of 0.4 (mass ratio). PC was substituted with VA by 20%, 30%, 40%, and 50% by mass of the cement. The mixture proportion and designated ID used in this study are summarized in Table 3. Powder (PC and VA) and tap water were mixed for 5 min under a laboratory condition (25 °C) and poured into cubical molds with dimensions of 50 × 50 × 50 mm thereafter. The samples were kept in a sealed condition for 24 h using plastic wraps before the samples were placed in an accelerated carbonation chamber. It should be noted that the samples for chemical analyses were crushed to pass through a 3 mm sieve before carbonation to achieve uniform carbonation. The samples were carbonation-cured at a 5% atmospheric CO_2_ concentration, 25 °C, and 60% R.H., until 28 days. Similarly, reference samples were cured for an identical period, in a sealed-condition using plastic wraps (i.e., free from carbonation), and placed in a thermo-hygrostat chamber (25 °C).

### 2.2. Test Methods

The compressive strength and CO_2_ uptake of carbonation-cured PC blended with VA were evaluated by means of compressive strength tests, XRD, and thermogravimetry/derivative thermogravimetry (TG/DTG). The samples were immersed in acetone at 7 and 28 days to arrest further evolution of hydrates. XRD and TG were conducted for the samples obtained at 7 and 28 days of curing, while other tests were performed at 28 days only.

The compressive strength was measured using a 3000 kN compression testing machine with a constant loading speed of 0.02 mm/s. The compressive strength was averaged from three replicas. An empyrean instrument with CuK*α* radiation at 40 kV and 30 mA was adapted for high-resolution XRD analysis. Samples were scanned in a range of 5–65° 2*θ* at a rate of 1°/min. The TG/DTG was performed using a TGA/DSC1/1600 LF instrument manufactured by Mettler-toledo (Columbus, OH, USA). The mass change was recorded under a constant heating rate of 10 °C/min in N_2_. 

## 3. Results

### 3.1. Compressive Strength

**Figure 3:**
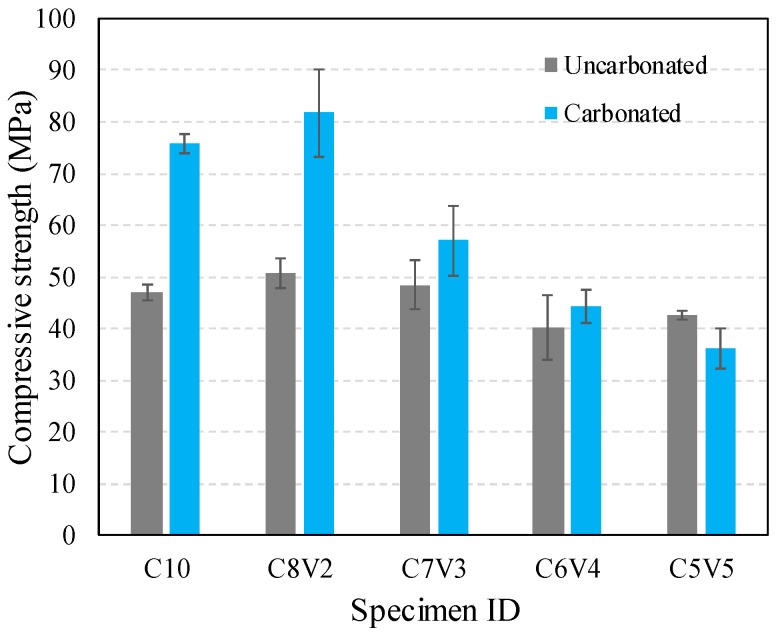
Compressive strength of normally cured and carbonation-cured volcanic ash-blended PC at 28 days.

The compressive strength of VA-blended PC is depicted in Figure 3. The compressive strength of uncarbonated samples varied depending on the amount of substituted VA. An increase in the compressive strength was most noticeable for the samples with 20% substitution with VA. Meanwhile, the samples incorporating more than 30% of VA showed compressive strength lower than that of the control specimen (i.e., C10).

Upon carbonation curing, most samples showed a significant increase in the compressive strength. Compared to uncarbonated samples, carbonation lead to an increase in strength that was highest for C8V2, and the strength decreased as the content of VA in the sample increased. In particular, carbonation curing of the samples blended with 50% VA was observed to reduce its strength. Overall, the compressive strength of carbonation-cured samples exhibited a similar trend with that of uncarbonated samples, showing a decrease as the amount of VA increased. It should be mentioned that the strength increases in the normally cured C8V2 and C7V3 samples compared to the C10 sample is possibly attributed to the filler effect of VA [40]. Also note that the strength increase in the carbonation-cured samples is mainly associated with the increased hydration reaction of PC promoted by carbonation curing. However, the decrease in the compressive strength of these samples is possibly attributed to the dilution effect, which occurs when the incorporated mineral admixture significantly influences the water-to-cement ratio [41]. Therefore, a long term compressive strength test is required to assess the effect of the pozzolanic reaction degree on the compressive strength.

### 3.2. XRD

**Figure 4:**
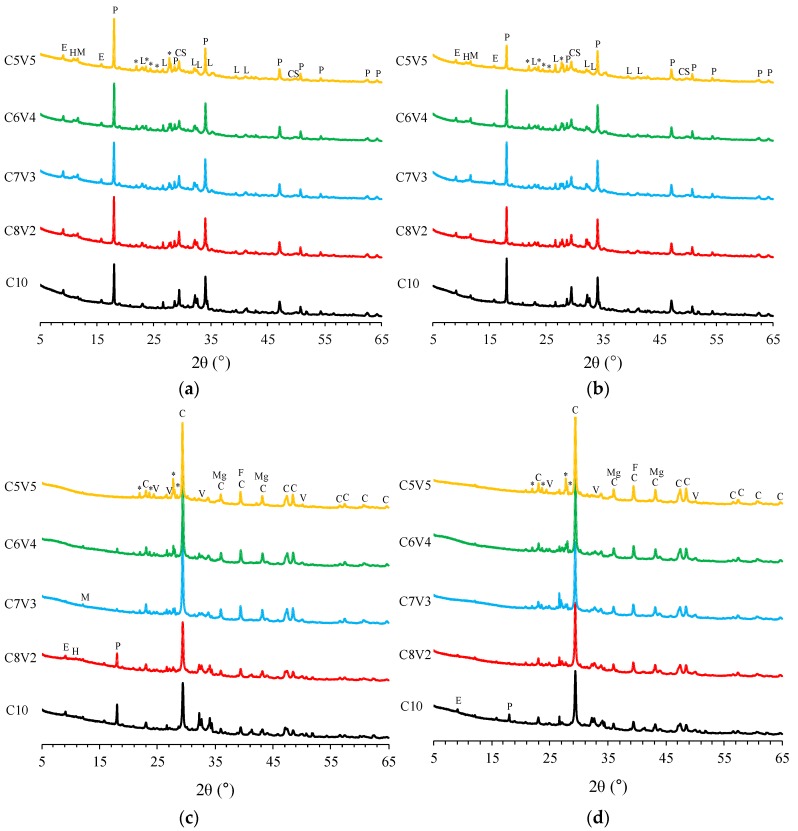
XRD patterns of normally cured and carbonation-cured volcanic ash-blended PC: (**a**) Normally cured 7 days; (**b**) normally cured 28 days; (**c**) carbonation-cured 7 days; (**d**) carbonation-cured 28 days. P: Portlandite, CS: C-S-H, L: Larnite, E: Ettringite, H: Hemicarbonate, M: Monocarbonate, C: Calcite, V: Vaterite, Mg: Dolomite, F: Siderite, and *: Andesine.

The XRD patterns of VA-blended PC are shown in Figure 4. The uncarbonated samples showed peaks corresponding to the presence of portlandite (Ca(OH)_2_, PDF #00-044-1481), C-S-H (Ca_1.5_SiO_3.5_H_2_O, PDF #00-033-0306), and larnite (Ca_2_SiO_4_, PDF #01-077-2010). The presence of ettringite (Ca_6_Al_2_(SO_4_)_3_(OH)_12_·_26_H_2_O, #PDF 00-013-0350) was commonly observed in the uncarbonated samples with several peaks attributed to andesine, which correspond to the unreacted fractions of raw volcanic ash. The formation of AFm phases, i.e., hemicarbonate (Ca_4_Al_2_O_7_(CO_2_)_0.5_·12H_2_O, PDF #00-036-0129) and monocarbonate (Ca_4_Al_2_O_7_(CO_2_)·11H_2_O, PDF #00-036-0377)), was observed in the VA-blended samples. The formation of these phases is possibly attributed to the use of partially carbonated VA given that Ca–, Mg–, and Fe– carbonates can be precipitated in natural basaltic VA due to weathering carbonation [42]. It should be noted that the peak associated with portlandite was observed even in the samples blended with 50% VA at 28 days. Note that portlandite is found to be fully consumed in PC blended with 20% fly ash or silica fume according to the thermodynamic calculation [11], indicating that the pozzolanic reaction of VA was relatively low.

The carbonation-cured samples showed peaks associated with the presence of calcium carbonate polymorphs, such as calcite (CaCO_3_, PDF #01-072-1937) and vaterite (CaCO_3_, #PDF 00-001-1033). In addition, peaks corresponding to the carbonation products of VA can be identified from the presence of siderite (FeCO_3_, PDF #00-029-0696) and dolomite (CaMg(CO_3_)_2_, #PDF 00-036-0426). At 7 days of carbonation curing, the C10 and C8V2 samples showed a peak corresponding to portlandite. The intensity of the peak corresponding to portlandite was found to decrease as the amount of VA substitution increased. This shows that carbonation had fully occurred in the samples blended with 50% VA. At 28 days of carbonation curing, the presence of ettringite and portlandite was not observed in the XRD patterns of VA-blended samples except for C10. In addition, peaks corresponding to the presence of hemicarbonate and monocarbonate disappeared at 28 days. The intensity of the peaks corresponding to andesine was persistent at 28 days, indicating that this phase contained in the raw VA remains unaffected by carbonation.

### 3.3. TG/DTG

**Figure 5:**
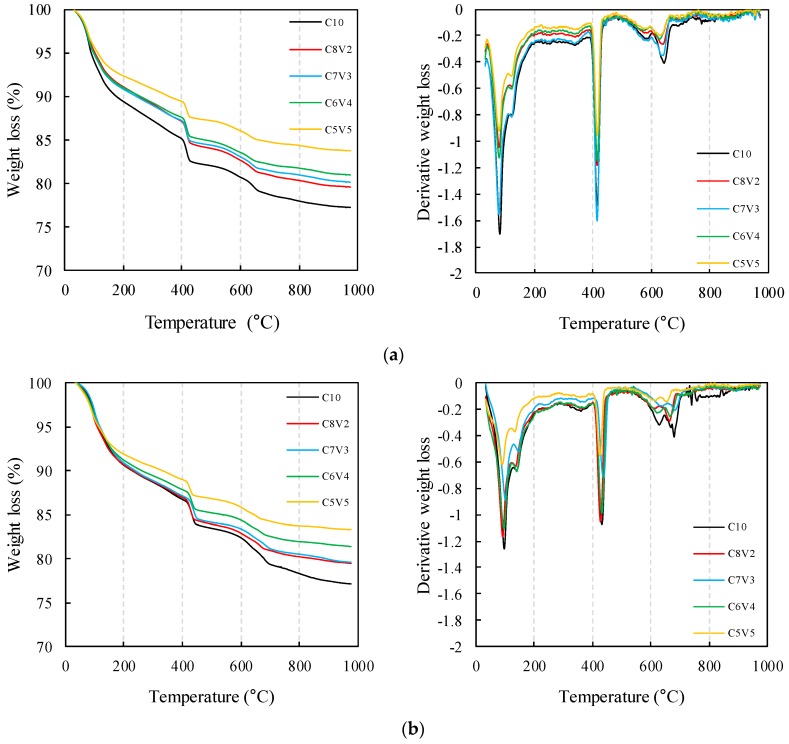

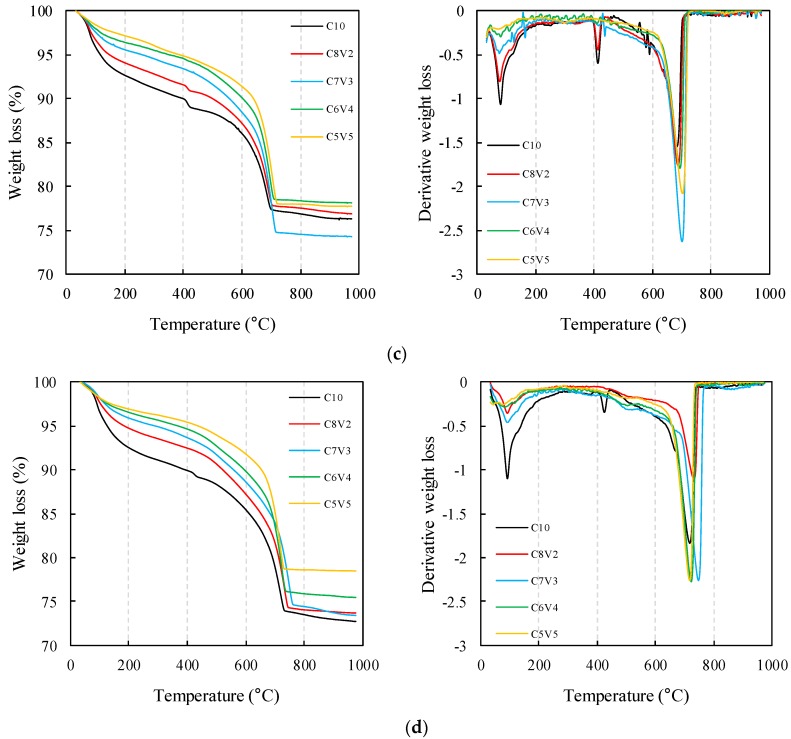
Thermogravimetry/derivative thermogravimetry (TG/DTG) curves of normally cured and carbonation-cured volcanic ash-blended PC: (**a**) Normally cured 7 days; (**b**) normally cured 28 days; (**c**) carbonation-cured 7 days; (**d**) carbonation-cured 28 days.

The TG/DTG curves of VA-blended PC are shown in Figure 5. The uncarbonated samples showed a weight loss peak at 80–100 °C and a shoulder at 120–140 °C due to the dehydration of chemically bound water from C–S–H [43] and ettringite [44]. The VA-blended samples showed a lesser weight loss at this region, implying that the amount of hydration products (C–S–H and ettringite) was much less. The weight loss at 400–450 °C is attributed to the dehydroxylation of portlandite [45], and was observed in all samples at seven and 28 days regardless of the amount of VA substitution. This is in fair agreement with the XRD results, showing that the pozzolanic reactivity of the VA was low. The weight loss at 500–720 °C can be attributed to the decarbonation of calcium carbonate [46] and unreacted clinker [47], and its intensity reduced with the amount of cement replacement.

The carbonation-cured samples showed a weight loss at regions corresponding to the dehydration of chemically bound water from hydration products [43,44] and dehydroxylation of portlandite [45], which is similar with the uncarbonated samples, but with reduced intensity. Meanwhile, a weight loss at 500–720 °C was observed in all samples at seven and 28 days, which is attributed to the decarbonation of calcium carbonate [46], mainly calcite, as observed by the XRD analysis. It is worth noting the carbonation-cured reference sample (i.e., without VA) showed a weight loss due to dehydroxylation of portlandite, while portlandite was found to have been fully consumed in the VA-blended samples at 28 days of carbonation curing due to their lower PC content, resulting in a rapid carbonation. The relative weight loss attributed to dehydration of C–S–H and ettringite, dehydroxylation of portlandite, and decarbonation of calcium carbonate of the samples at 28 days as calculated by TG is summarized in Table 4.

The CO_2_ uptake capacity per unit weight of cement in the total blend at 28 days of carbonation curing, relative to the unit weight of PC, is summarized in Table 5, which is calculated by the following Equation (1) [14]:

CO_2_ uptake capacity (%) = M_CO2_/M_PC_ × 100 (1)

**Figure 6:**
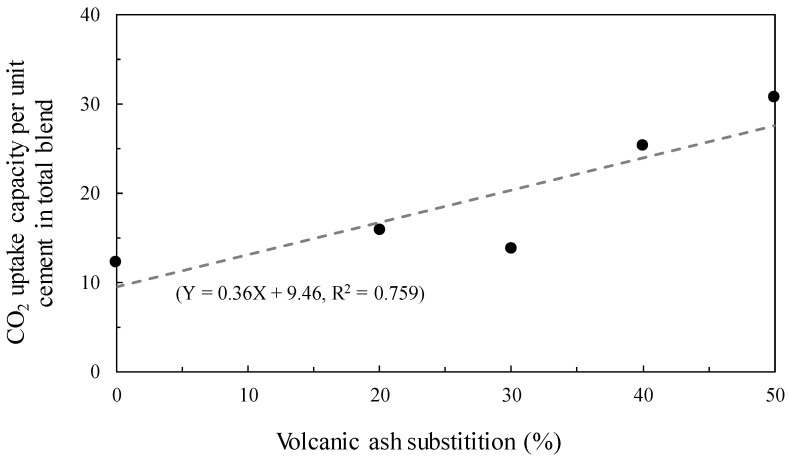
Relationship between CO_2_ uptake capacity per unit cement in the total blend of volcanic ash-blended PC and amount of volcanic ash substitution.

Here, M_CO2_ is the mass of CO_2_ sequestrated by PC during the carbonation curing, and is calculated by computing the difference between the weight loss at 500–720 °C of carbonation-cured and uncarbonated samples. M_PC_ is the unit mass ratio of the PC used (i.e., 0.71, 0.57, 0.5, 0.43, and 0.36 for C10, C8V2, C7V3, C6V4, and C5V5 samples, respectively). The CO_2_ uptake capacity of the reference sample was 12.3%, showing a similar level of CO_2_ uptake (13.5%) observed in a previous study [14]. The relationship between the CO_2_ uptake capacity of VA-blended PC and VA substitution is correlated in Figure 6. The CO_2_ uptake capacity of VA-blended PC was found to generally increase with the amount of VA substitution.

## 4. Discussion

The obtained results can be discussed in terms of two main roles of VA blended in PC, namely hydration and carbonation. AFm phases, such as hemicarbonate and monocarbonate, were observed in the VA-blended samples, which can be attributed to the incorporation of SCM rich in Al, as typically found in fly ash-blended cement [11]. The DTG results showed that a significant amount of portlandite remained unconsumed in the samples blended with a high amount of VA (i.e., 50%), indicating that the dilution effect occurred in the samples and that 28 days was insufficient for the VA to exhibit pozzolanic reaction. This is also supported by the fact that the weight loss due to the dehydration of hydration products in the DTG curve was relatively lower in these samples. 

Considering the fact that the CO_2_ uptake capacity of cementitious materials heavily depends on their Ca content, which effectively facilitates as a CO_2_ sink by precipitation of CaCO_3_ polymorphs [13], one may assume that incorporation of SCMs with a low content of Ca may lead to a lower CO_2_ uptake in the cementitious material. The amount of CO_2_ in the samples after 28 days of carbonation curing ranged from 6.9–11.0%, showing a large deviation. However, the CO_2_ uptake capacity relative to the unit mass ratio of PC approximately showed a linear increase with the amount of VA substitution, meaning that the CO_2_ uptake efficiency can be vastly improved by VA substitution. Furthermore, the relative amount of stored CO_2_ (fourth column of Table 5), without consideration of unit cement in total blend, also demonstrated that incorporated VA increases CO_2_ diffusivity even in samples with less PC content. This may be attributed to the presence of CaO, Fe_2_O_3_, and MgO components in VA, which amounts to 23.87 wt.%, precipitating carbonates in addition to the carbonation of PC. The traces of these carbonates were observed in the XRD patterns of carbonated samples, which are overlapped with the peaks corresponding to calcite. However, the presence of various carbonates is supported by the fact that the peak intensities corresponding to these carbonates increased as the amount of incorporated VA increased. VA incorporation in PC can therefore be described to promote the diffusion of CO_2_ into a cementitious matrix, thereby increasing the rate of carbonation, as suggested by the higher compressive strength being reached by VA-blended samples (Figure 3). In light of the industrial practice of carbonation curing for concrete production, VA incorporation is effective to lower the overall CO_2_ footprint by reducing the use of PC and enhancing the CO_2_ uptake efficiency of the cement used. In particular, these advantageous aspects are considered to be very useful when VA blended PC is used in the production of precast members.

## 5. Conclusions

The present study investigated the CO_2_ uptake capacity of carbonation-cured cement blended with ground VA. Samples at various blend ratios were manufactured and carbonation-cured for 28 days. The results obtained in this study showed that carbonation curing of VA incorporated cement exhibited a significant enhancement in the compressive strength, thus enabling a reduction in the CO_2_ footprint. The following conclusions were drawn from this work:

(1) A significant enhancement in the compressive strength by carbonation curing was observed in the VA-blended cement except for the samples blended with more than 40% VA, which were found to be fully carbonated.

(2) The CO_2_ uptake capacity of VA-blended samples relative to the unit weight of PC showed a linear increase as the amount of VA substitution increased due to the partial carbonation of VA.

(3) VA incorporation of cement can significantly reduce the CO_2_ footprint throughout its production and manufacturing, and is capable of improving CO_2_ diffusivity.

(4) The utilization of VA as a SCM has a slight effect in improving the compressive strength of PC, although the dilution effect somewhat hindered the strength development of the samples blended with VA. Nevertheless, combining VA substitution with ACC resulted in a significant improvement in the compressive strength and CO_2_ uptake capacity.

## Figures and Tables

**Table 1:** Chemical composition of the Portland cement.

Chemical Compound (%)	Portland Cement
CaO	63.84
SiO_2_	21.45
Al_2_O_3_	6.03
Fe_2_O_3_	3.27
SO_3_	2.13
L.O.I. ^1^	3.27

^1^ Loss-on-ignition.

**Table 2:** Chemical composition of the volcanic ash.

Chemical Compound (%)	Volcanic Ash
Na_2_O	5.08
MgO	3.20
Al_2_O_3_	17.30
SiO_2_	48.10
P_2_O_5_	0.55
SO_3_	0.20
Cl	0.15
K_2_O	2.18
CaO	7.57
TiO_2_	2.41
MnO	0.26
Fe_2_O_3_	13.10

**Table 3:** Mixture proportion of samples expressed as mass ratio.

Specimen ID ^1^	Cement	Volcanic Ash	Water/powder ^2^ Ratio
C10	1	0.0	0.4
C8V2	0.8	0.2	0.4
C7V3	0.7	0.3	0.4
C6V4	0.6	0.4	0.4
C5V5	0.5	0.5	0.4

^1^ The following denotation was used to identify samples: “C” and “V” indicate Portland cement (PC) and volcanic ash (VA)., respectively, while the numbers following “C” and “V” indicate their mass ratio. ^2^ Powder denotes the summation of the amount of cement and volcanic ash.

**Table 4:** Relative weight loss (%) attributed to the decomposition of reaction products at 28 days.

Curing Condition	Specimen ID	C-S-H and Ettringite (50–200 °C)	Portlandite (400–450 °C)	Calcium Carbonate (500–720 °C)
Uncarbonated	C10	9.16	2.83	4.31
	C8V2	8.94	2.59	3.18
	C7V3	8.91	2.56	3.19
	C6V4	8.34	2.28	2.82
	C5V5	7.47	1.86	2.72
Carbonated	C10	7.20	0.88	13.1
	C8V2	5.06	0.69	12.3
	C7V3	3.93	0.79	10.3
	C6V4	3.05	0.76	13.7
	C5V5	2.59	0.62	13.7

**Table 5:** CO_2_ uptake capacity of carbonation-cured volcanic ash-blended cement calculated from thermogravimetry results (%).

Specimen ID	M_carbonation-cured_ (a)	M_uncarbonated_ (b)	(a–b)	CO_2_ Uptake Capacity Per Unit Cement in Total Blend
C10	13.1	4.3	8.8	12.3
C8V2	12.3	3.2	9.1	15.9
C7V3	10.1	3.2	6.9	13.8
C6V4	13.7	2.8	10.9	25.4
C5V5	13.7	2.7	11.0	30.7
